# Supplementary material for: The Combination of Patient-Reported Clinical Symptoms and an Endoscopic Score Correlates Well with Health-Related Quality of Life in Patients with Ulcerative Colitis
Source: J Clin Med. 2019 Aug 5;8(8):1171. doi: 10.3390/jcm8081171 (PMC6723355; doi:10.3390/jcm8081171)
Supplement: Supplementary file 1 [file jcm-08-01171-s001.pdf]

Supplementary table 1: Patient-reported clinical outcomes using the 6-point partial Mayo score

Circle one number which indicates your symptoms in the **past 7 days**

**Rectal bleeding**	
**0**	no blood seen
**1**	streaks of blood < 50%
**2**	obvious blood > 50%
**3**	blood passes alone

**Stool frequency**	
**0**	normal
**1**	1-2 per day more than normal
**2**	3-4 per day more than normal
**3**	5^+ per day more than normal

Supplementary table 2: Short Inflammatory Bowel Disease Questionnaire (SIBDQ)

Circle a number which indicates how you have been feeling during **the last 2 weeks.**

1. How often has **the feeling of fatigue or being tired and worn out** been a problem for you during the past 2 weeks?

1	2	3	4	5	6	7
All of the time	Most of the time	A good bit of the time	Some of the time	A little of the time	Hardly any of the time	None of the time

2. How often during the last 2 weeks have you **delayed or canceled a social engagement** because of your bowel problem?

1	2	3	4	5	6	7
All of the time	Most of the time	A good bit of the time	Some of the time	A little of the time	Hardly any of the time	None of the time

3. As a result of your bowel problems, how much difficulty did you experience **doing leisure or sports activities** you would like to have done during the past 2 weeks?

1	2	3	4	5	6	7
A great deal of difficulty	A lot of difficulty	A fair bit of difficulty	Some difficulty	A little difficulty	Hardly any difficulty	No difficulty

4. How often during the past 2 weeks have you been **troubled by pain in the abdomen**?

1	2	3	4	5	6	7
All of the time	Most of the time	A good bit of the time	Some of the time	A little of the time	Hardly any of the time	None of the time

5. How often during the past 2 weeks have you **felt depressed or discouraged**?

1	2	3	4	5	6	7
All of the time	Most of the time	A good bit of the time	Some of the time	A little of the time	Hardly any of the time	None of the time

6. Overall, in the past 2 weeks, how much of a problem have you had with **passing large amounts of gas**?

1	2	3	4	5	6	7
Major problem	A big problem	A significant problem	Some problem	A little trouble	Hardly any trouble	No trouble

7. Overall, in the past 2 weeks, how much of a problem have you had **maintaining or getting to the weight** you would like to be?

1	2	3	4	5	6	7
Major problem	A big problem	A significant problem	Some problem	A little trouble	Hardly any trouble	No trouble

8. How often during the past 2 weeks have **you felt relaxed and free of tension**?

1	2	3	4	5	6	7
None of the time	Hardly any of the time	A little of the time	Some of the time	A good bit of the time	Most of the time	All of the time

9. How much of the time during the past 2 weeks have you been troubled by **a feeling of having to go to the bathroom even though your bowels were empty**?

1	2	3	4	5	6	7
All of the time	Most of the time	A good bit of the time	Some of the time	A little of the time	Hardly any of the time	None of the time

10. How often during the past 2 weeks have you **felt angry as a result of your bowel problem**?

1	2	3	4	5	6	7
All of the time	Most of the time	A good bit of the time	Some of the time	A little of the time	Hardly any of the time	None of the time

Supplementary table 3: European quality 5 dimensions 3 level questionnaire (EQ5D3L)

By placing a tick in one box in each group below, please indicate

which statements best describe your own health state **today**.

	**Mobility**
❑	I have no problems in walking about
❑	I have some problems in walking about
❑	I am confined to bed
	**Self-Care**
❑	I have no problems with self-care
❑	I have some problems washing or dressing myself
❑	I am unable to wash or dress myself
	**Usual Activities** (e.g. work, study, housework, family or leisure activities)
❑	I have no problems with performing my usual activities
❑	I have some problems with performing my usual activities
❑	I am unable to perform my usual activities
	**Pain/Discomfort**
❑	I have no pain or discomfort
❑	I have moderate pain or discomfort
❑	I have extreme pain or discomfort
	**Anxiety/Depression**
❑	I am not anxious or depressed
❑	I am moderately anxious or depressed
❑	I am extremely anxious or depressed

Supplementary table 4: Work Productivity and Activity Impairment Questionnaire (WPAI)

1. Are you **currently employed** (working for pay)? ❑ **NO** ❑ **YES**

   If NO, check "NO" and skip to question 6.

The next questions are about the **past 7 days**, not including today.

2. During the past 7 days, **how many hours did you miss from work because of problems associated with your PROBLEM**? *Include hours you missed on sick days, times you went in late, left early, etc., because of your PROBLEM. Do not include time you missed to participate in this study.* ________**HOURS**

3. During the past 7 days, **how many hours did you miss from work because of any other reason**, such as vacation, holidays; time off to participate in this study? _________**HOURS**

4. During the past 7 days, **how many hours did you actually work**? _____**HOURS** *(If "0", skip to question 6.)*

5. During the past 7 days, **how much did your PROBLEM affect your productivity while you were working**?

If PROBLEM affected your work only a little, choose a low number.

PROBLEM had **no effect** on my work	0	1	2	3	4	5	6	7	8	9	10	PROBLEM **completely prevented me** from working

6. During the past 7 days, how much did **your PROBLEM affect your ability to do your regular daily activities, other than work at a job**?

If PROBLEM affected your activities only a little, choose a low number.

PROBLEM had **no effect** on my daily activities	0	1	2	3	4	5	6	7	8	9	10	PROBLEM **completely prevented me** from doing my daily activities

Supplementary table 5: Functional Assessment of Chronic Illness Therapy-Fatigue Scale (FACIT-F)

Below is a list of statements that other people with your illness have said are important. Please circle or mark one number per line to indicate your response as it applies to **the past 7 days**

	**Not at all**	**A little bit**	**Some-what**	**Quite a bit**	**Very much**
I feel fatigued...	0	1	2	3	4
I feel weak all over..	0	1	2	3	4
I feel listless (“washed out”)..	0	1	2	3	4
I feel tired..	0	1	2	3	4
I have trouble starting things because I am tired.......................	0	1	2	3	4
I have trouble finishing things because I am tired.....................	0	1	2	3	4
I have energy..	0	1	2	3	4
I am able to do my usual activities..	0	1	2	3	4
I need to sleep during the day..	0	1	2	3	4
I am too tired to eat..	0	1	2	3	4
I need help doing my usual activities..	0	1	2	3	4
I am frustrated by being too tired to do the things I want to do...	0	1	2	3	4
I have to limit my social activity because I am tired.................	0	1	2	3	4

Supplementary table 6: Hospital Anxiety and Depression Scale (HADS)

Please circle a number that is closest to how you have been feeling in **the past 7 days.**

**I feel tense or 'wound up':**		**I feel as if I am slowed down:**	
Most of the time	3	Nearly all of the time	3
A lot of the time	2	Very often	2
Time to time, occasionally	1	Sometimes	1
Not at all	0	Not at all	0
**I still enjoy the things I used to enjoy:**		**I get a sort of frightened feeling like 'butterflies in the stomach':**	
Definitely as much	0	Not at all	0
Not quite so much	1	Occasionally	1
Only a little	2	Quite often	2
Not at all	3	Very often	3
**I get a sort of frightened feeling like something awful is about to happen:**		**I have lost interest in my appearance:**	
Very definitely and quite badly	3	Definitely	3
Yes, but not too badly	2	I don't take as much care as I should	2
A little, but it doesn't worry me	1	I may not take quite as much care	1
Not at all	0	I take just as much care as ever	0
**I can laugh and see the funny side of things:**		**I feel restless as if I have to be on the move:**	
As much as I always could	0	Very much indeed	3
Not quite so much now	1	Quite a lot	2
Definitely not so much now	2	Not very much	1
Not at all	3	Not at all	0
**Worrying thoughts go through my mind:**		**I look forward with enjoyment to things:**	
A great deal of the time	3	A much as I ever did	0
A lot of the time	2	Rather less than I used to	1
From time to time but not too often	1	Definitely less than I used to	2
Only occasionally	0	Hardly at all	3
**I feel cheerful:**		**I get sudden feelings of panic:**	
Not at all	3	Very often indeed	3
Not often	2	Quite often	2
Sometimes	1	Not very often	1
Most of the time	0	Not at all	0
**I can sit at ease and feel relaxed:**		**I can enjoy a good book or radio or TV program:**	
Definitely	0	Often	0
Usually	1	Sometimes	1
Not often	2	Not often	2
Not at all	3	Very seldom	3
